# Role of Succinate Dehydrogenase in Age‐Related Th17 Inflammation

**DOI:** 10.1111/acel.70451

**Published:** 2026-03-24

**Authors:** Evelyn Ocegueda, Gabrielle Chase, Michaella Niceforo, Aida Javidan, Lydia Gugliuzza, Jingting Yu, Rachel Y. Kang, Ava Lankowski, Olivia Stefanik, Kailey Leclerc, Yolander Valentine, Micah J. Drummond, Jude T. Deeney, Josephine S. Modica‐Napolitano, Elizabeth A. Proctor, Hatice Hasturk, Barbara S. Nikolajczyk, Leena P. Bharath

**Affiliations:** ^1^ Department of Health Sciences and Nutrition Merrimack College North Andover Massachusetts USA; ^2^ Department of Natural Sciences Merrimack College North Andover Massachusetts USA; ^3^ Department of Pharmacology and Nutritional Sciences University of Kentucky Lexington Kentucky USA; ^4^ Razavi Newman Integrative Genomics and Bioinformatics Core The Salk Institute for Biological Studies La Jolla California USA; ^5^ Department of Neurosurgery Penn State College of Medicine Hershey Pennsylvania USA; ^6^ Department of Neuroscience & Experimental Therapeutics Penn State College of Medicine Hershey Pennsylvania USA; ^7^ Department of Physical Therapy and Athletic Training University of Utah Salt Lake City Utah USA; ^8^ Department of Endocrinology, Diabetes, Nutrition & Weight Management Boston University School of Medicine Boston Massachusetts USA; ^9^ Department of Biomedical Engineering, Penn State Neuroscience Institute Pennsylvania State University University Park Pennsylvania USA; ^10^ Department of Engineering Science & Mechanics, Penn State Neuroscience Institute Pennsylvania State University University Park Pennsylvania USA; ^11^ ADA Forsyth Institute Somerville Massachusetts USA; ^12^ Barnstable Brown Diabetes and Obesity Center University of Kentucky Lexington Kentucky USA

**Keywords:** Aging, Complex II, Cytokines, Mitochondria, SDH, T cells, Th17 cytokines

## Abstract

Age‐related cellular changes negatively impact CD4^+^ T cell function. Our prior work showed that mitochondrial complex II (succinate dehydrogenase [SDH]) expression was upregulated in T cells from older (O) adults (60–80 years old). T cells from older adults also produced higher amounts of cytokines generally considered proinflammatory, such as Th17 cytokines IL‐17A/F and IL‐21, and the Th‐17‐supportive cytokine IL‐6, compared to T cells from younger (Y) adults (25–40 years old). The objective of our study is to evaluate whether hyperactivation of SDH is required for the induction of proinflammatory cytokines and the mechanistic link between SDH and Th17 cytokine production. CD4^+^ T cells were isolated from lean normoglycemic younger (avg: 31.58 years; BMI 21.14 kg/m^2^) and older (avg: 64.81 years; BMI 21.95 kg/m^2^) adults. SDH was pharmacologically and genetically modulated, and mitochondrial structure, function, metabolites, and cytokine production were quantified. SDH activation in T cells from older adults induced heightened oxidation of succinate, disrupted the fumarate‐to‐succinate ratio, stabilized HIF‐1α, and promoted Th17 cytokines. Genetic and pharmacological inhibition of SDH in T cells from older adults lowered proinflammatory cytokine production, whereas exogenous addition of cell‐permeable succinate induced SDH protein in T cells from younger adults and recapitulated the proinflammatory Th17 profile observed in T cells from older adults. These data establish a mechanistic link between SDH and Th17 inflammation.

## Graphical abstract

Age‐induced mitoSTAT3‐dependent upregulation of mitochondrial complex II; succinate dehydrogenase (SDH), in CD4^+^ T cells promotes an imbalance in the levels of metabolites succinate and fumarate, characterized by lower succinate and higher fumarate. Changes in the metabolites alter mitochondrial structure and function and promote HIF1α to induce the production of Th17 and Th17 supportive cytokines. Graphical abstract was created using https://bioart.niaid.nih.gov/.

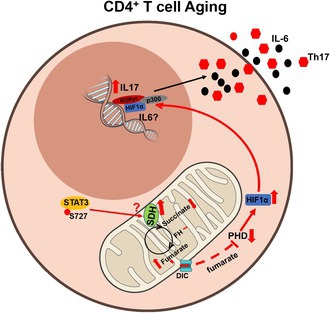

## 1 Introduction

The mechanistic regulation of the immune system by metabolites has gained much attention over the past decade. Many experimental studies focus on targeting metabolites and metabolic reprogramming as promising therapeutic strategies for immune‐mediated disorders (Pålsson‐McDermott and O'Neill 2020). As newer studies emerge, the role of metabolites in regulating many age‐related inflammatory conditions is becoming increasingly evident (Ginefra et al. 2024; Hanlon et al. 2022). Seminal work shows that metabolism intricately regulates functions of immune cells such as cytokine production (Lampropoulou et al. 2016), antibody production (Fu et al. 2022), differentiation (Kunisawa et al. 2015), and overall immune response (Scagliola et al. 2020).

CD4^+^ T cells (T cells herein) play a critical role in immune responses, acting as coordinating centers of the adaptive immune system and mediating protection against infection and malignancy. However, T cells are also implicated in aberrant immune responses, such as chronic inflammation (Goto et al. 2024; Shchukina et al. 2023; Bharath et al. 2020), autoimmunity (Goto et al. 2024), and are known to play a complex role in inflammaging (Shchukina et al. 2023; Bharath et al. 2020). Some studies have evaluated metabolites that regulate T cell function during inflammation. These include metabolic reprogramming upon activation of T cells, which triggers signal transduction and induces changes in the amounts of cellular metabolites (Ma et al. 2024; Bailis et al. 2019).

Succinate dehydrogenase (SDH; mitochondrial complex II) bridges the tricarboxylic acid (TCA) cycle and oxidative phosphorylation (OXPHOS) by enzymatically converting the dicarboxylic acid succinate to fumarate while simultaneously reducing FAD to FADH2 and transferring the electrons through the electron transport chain, subsequently reducing ubiquinone to ubiquinol and contributing to ATP synthesis (Rustin et al. 2002). Despite being the smallest respiratory complex, SDH, comprising four subunits, regulates several physiological processes. Dysregulation of SDH can lead to succinate accumulation, which acts as an oncometabolite and promotes inflammation (Huang et al. 2024). Inherited SDH mutations cause early‐onset encephalomyopathy (Leigh syndrome), paraganglioma, susceptibility to tumors, and optic atrophy (Liu et al. 2023).

Our prior work showed that T cells from older adults have higher SDH transcript levels and produce more Th17 cytokines when compared to T cells from younger adults (Zukowski et al. 2023). T cells from older adults also exhibited mitochondrial structural and functional characteristics distinct from those of younger adults' T cells. Our work identified that the age‐related mitochondrial phenotype was secondary to increased mitochondrial localization of serine 727‐phosphorylated Signal Transducer and Activator of Transcription 3 (STAT3). When a mitochondria‐targeted STAT3 inhibitor displaced the mitochondrial localization of STAT3, age‐induced hyperactivation of SDH was prevented and Th17 cytokine production was reduced.

In this study, we tested the hypothesis that hightened SDH activation is required for the production of Th17 cytokines during aging. Additionally, we evaluated the mechanism(s) through which SDH promoted Th17 inflammation during aging. Our data show that pharmacological and genetic inhibition of SDH in T cells from older adults restored mitochondrial structure and function to levels approximating those of T cells from younger adults. SDH inhibition also lowered Th17 cytokines and promoted IL‐4 production. We identified lower cellular succinate, resulting from heightened SDH activation, an ensuing imbalance in the succinate‐to‐fumarate ratio, and subsequent stabilization of HIF‐1α as the mechanistic regulator of Th17 cytokines during aging. Here, we define a previously unappreciated mechanism that promotes Th17 inflammation in lean, normoglycemic older adults, linked to mitochondrial complex II (SDH).

## 2 Materials and Methods

### 2.1 Human Sample Collection

In accordance with the Declaration of Helsinki, informed consent from all human participants was obtained following Institutional Review Board‐approved protocols (protocol no. 21‐01) at the ADA Forsyth Institute. Peripheral blood was obtained approximately during midday from unfasted normoglycemic younger adults who were lean (Y; range: 26–38 years old; BMI < 25 kg/m^2^) or normoglycemic older adults who were lean (O; range: 60–72 years old; BMI < 25 kg/m^2^). Metabolic health was assessed by HbA1C measured by a standard commercial laboratory test (Quest Diagnostics, MA). Patient characteristics are shown in Table 1. Exclusion criteria were smoking within the past 12 months, recent use of antibiotics or anti‐inflammatory medications, that is, NSAIDs/steroids, colds/flu/COVID‐19 within the past 2 weeks, BMI > 25 kg/m^2^, type 2 diabetes or diabetes medications, and any history of cancer, hyperglycemia, and autoimmune diseases. Blood samples were also obtained from a commercial vendor based on the above‐mentioned inclusion and exclusion criteria (Biocollections Worldwide Inc., Miami, FL).

**TABLE 1:** Description of research subjects.

	Young	Old
Total *N*	12	16
Age, years (mean [range])*	31.58 (26–38)	64.81 (60–72)
BMI, kg m^−2^ (mean [range])	21.14 (18.80–23.30)	21.95 (19.10–24.90)
Females (*N* [%])	5 (41.66%)	10 (62.50%)
Males (*N* [%])	7 (58.33%)	6 (37.50%)

*Note:* * *p* < 0.05.

### 2.2 Isolation of PBMCs and T Cells

Peripheral blood mononuclear cells (PBMCs) and CD4^+^ T cells were isolated according to our established protocol (Conway et al. 2022; Bharath et al. 2020; Zukowski et al. 2023). Briefly, 50 mL of peripheral blood was collected into acid/citrate/dextrose containing tubes by venous puncture. PBMCs were purified by Histopaque. CD4^+^ T cells were isolated from the PBMCs by negative selection using MACS columns (Miltenyi Biotech). Isolated cells were placed in −80°C for at least 24 h and then stored at −190°C in liquid nitrogen.

### 2.3 Cell Treatment

CD4^+^ T cells obtained from lean younger and older adults were stimulated in vitro for 40 h with T cell‐activating CD3/CD28 Dynabeads (Thermo Fisher Scientific, 11132D) at 2 μL Dynabeads per 100k cells. Concurrent with CD3/CD28 activation, the cells were treated with the following treatments, One millimolar (1 mM) 3‐nitropropionic acid (3NP), an inhibitor of SDH (MedChem Express, Monmouth Junction, NJ), or 5 mM diethyl succinate (DES), a cell‐permeable form of succinate (Millipore Sigma, Burlington, MA) (Bailis et al. 2019). DES and 3NP added along with CD3/CD28 remained in the culture for 40 h. In some experiments, cells were treated with mitochondria‐targeted superoxide dismutase mimetic and ROS scavenger 10 μM Mitotempo (MTTempo) or 10 μM Tempol (Tempol) (Millipore Sigma, Burlington, MA) 3 h post‐activation, and remained in the culture for 37 h. Our prior experimental evaluation showed that the addition of ROS inhibitors/scavengers, along with activation, decreased cell viability. Thus, our standard protocol for adding ROS scavengers is 3 h post‐activation. In some experiments, cell cultures were treated with 1 μM scrambled control siRNA or SDHA siRNA (Dharmacon, Lafayette, CO). In some experiments, cell cultures were treated with 1 μM fumarate hydratase (FH) inhibitor, FH‐IN‐1 (MedChem Express) for 40 h (Yan et al. 2024; Hooftman et al. 2023). All treatments were added to our standard culture media of RPMI with 5 mM glucose (normoglycemic), 1 mM sodium pyruvate, 10% heat‐inactivated FBS, and antibiotics. Accell siRNA transfection media (Dharmacon, Lafayette, CO) was used for transfection of the siRNA. Supernatants were collected and stored at −80°C and were used for ELISA or were shipped on dry ice to the University of Kentucky for bioplex assay to quantify cytokines.

### 2.4 Immunofluorescence

CD4^+^ T cells were collected from younger and older adults after SDH modulation as described above. The cells were collected and plated onto poly‐D‐lysine‐coated coverslips in 12‐well plates. The cells were briefly centrifuged (1200 rpm, 10 min), washed two times with 1× PBS and incubated in 4% paraformaldehyde for 15 min at RT. The coverslips were washed 2× with PBS and 0.1% triton X‐100 (PBST), and were blocked for at least 30 min in 2% BSA/PBST. Antibodies to SDHA (Cell Signaling Technology, Danvers, MA), SDHB, TOM20, and FIS1 (Santacruz Biotechnology, Dallas, TX) were added at a 1:50 dilution with incubation overnight at 4°C. The coverslips were washed 2× with PBST and incubated with fluorophore‐tagged secondary antibodies at 1:500 (anti‐mouse Alexa 488 or anti‐rabbit Alexa 647) (Rockland Immunochemicals, Limerick, PA) for 2 h at RT. The coverslips were washed 2× with PBST and mounted on glass slides using Fluoromount G (Southern Biotech, Birmingham, AL). Cell imaging was performed using a 63× oil‐immersion lens on a Zeiss LSM 800 confocal microscope. Gain was adjusted independently for each fluorophore while avoiding saturation and keeping the laser power as low as possible. The range indicator was used to avoid saturation and checked every time an image was acquired. The images were acquired to use the full dynamic range without saturating the pixels or losing weak signal‐to‐noise. We performed a 16‐bit acquisition, and the settings were kept identical throughout imaging for each experiment and across all treatments. Approximately 5–7 fields/slide were imaged in *N* = 3–4 subjects per treatment, and data were analyzed using FIJI/ImageJ. Microscopy images(blinded) were processed and analyzed as described (Valente et al. 2017; Kirber et al. 2007). Manual segmentation was performed on the images, carefully avoiding cells without clear boundaries, and regions of interest (ROI) were obtained. Background correction was performed and globally applied to all images from all the donors. For colocalization analysis, Costes' automatic thresholding was performed. Protein expression was reported as mean fluorescence intensity (MFI), and colocalization between two proteins was reported as Pearson's Colocalization Coefficient (PCC). PCC is a well‐established method that quantifies the degree of overlap between fluorescence in the channels. PCC is unaffected by changes in the offset and independent of gain (Zukowski et al. 2023; Adler and Parmryd 2010).

### 2.5 Immunoblotting

Immunoblotting quantified protein expression as we published (Conway et al. 2022; Bharath et al. 2020; Zukowski et al. 2023). Briefly, 30 μL of 1× cell lysis buffer (Cell Signaling Technology, Danvers, MA) was added to 1 × 10^6^ cells and incubated on ice for 20 min. Cells were centrifuged at 13,000 rpm for 20 min, and protein concentration in the supernatant was assessed using a Bicinchoninic acid assay (Thermo Fisher Scientific). An equal amount of protein (15 μg/well) was loaded into each well of polyacrylamide gels, and electrophoresis was performed at 150 V for 45 min. Transfer of protein to polyvinylidene difluoride (PVDF) membrane was performed at 35 V for 4 h. The membrane was blocked for 30 min at room temperature (RT) in blocking buffer containing 2% bovine serum albumin in TBST, followed by overnight incubation at 4°C in the respective primary antibodies. The membrane was washed 2× with 1× TBST, and incubated with the respective secondary antibodies for 2 h at RT, then imaged. Table 2 lists the antibodies used in this study. All primary antibodies were used at a dilution of 1:500. All secondary antibodies were used at a dilution of 1:20,000. Protein expression on Western blots was quantified using Image Studio software (Licor, Lincoln, NE). The expression levels of each protein of interest were normalized to the loading control, β‐actin, as per standard immunoblotting methods. The relative expression of the protein of interest in the control (O; T cells from older adults) was compared with the treatments O + 3NP and O + DES, and the young control (Y; T cells from younger adults) was compared with the treatment Fumarate hydratase inhibition Y + FHIN‐1.

**TABLE 2:** Resource identification reagents or resources.

Antibodies	Source	Identifier
FIS1	Santa Cruz Biotechnology	Cat no. sc‐376447, RRID:AB_11149382
VDAC1	Cell Signaling Technology	Cat no. 4866S, RRID: AB_2272627
Actin	Cell Signaling Technology	Cat no. 3700S, RRID: AB_2242334
SDHA	Santa Cruz Biotechnology	Cat no. sc‐390381, RRID: AB_476744
SDHA	Cell Signaling Technology	Cat# 11998S, RRID: AB_2750900
SDHB	Santa Cruz Biotechnology	Cat no. sc271548, RRID: AB_10659104
OGDH	Cell Signaling Technology	Cat no. 26865S, RRID: AB_2737585
HIF‐1α	Cell Signaling Technology	Cat no. 36169S, RRID: AB_2799095
Anti‐TOM20	Santa Cruz Biotechnology	Cat no. sc‐17764, RRID: AB_628381
Anti‐mouse Alexa 488 used at 1:500	Rockland antibodies	Cat no. 610‐741‐124, RRID: AB_1057558
Anti‐rabbit Alexa 647 used at 1:500	Thermo Fisher Scientific	Cat no. A‐21244, RRID: AB_2535812
Anti‐mouse IRDye used at 1:20,000	Licor	Cat no. 926‐32210, RRID: AB_621842
Anti‐rabbit IRDye used at 1:20,000	Licor	Cat no. 926‐68071, RRID: AB_10956166
*Biological samples*		
Lean normal glucose‐tolerant younger adults, CD4^+^ T cells Table 1	This paper	NA
Lean normal glucose‐tolerant older adults, CD4^+^ T cells Table 1	This paper	NA
*Chemicals, peptides, and recombinant proteins*		
Bovine serum albumin	Sigma Aldrich	Cat no. A8806
Antimycin A	Enzo Life Sciences	ALX‐380‐075‐M010
Oligomycin	Calbiochem	Cat no. 495455
FCCP	Enzo Life Sciences	Cat no. ML‐CM120‐0050
Rotenone	Enzo Life Sciences	Cat no. ALX‐350‐360‐G001
*Critical commercial assays*		
Milliplex human Th17 25‐plex kit	Millipore Sigma	Cat no. HT17MG‐14K‐PX25
Legend Max IL17 A/F ELISA	Biolegend	Cat no. 435807
EnzyChrom Succinate Assay Kit	Bio Assay Systems	Cat no. ESNT‐100
Fumarate Assay Kit	Abcam	Cat no. AB102516
Lactate Assay Kit	Sigma Aldrich	Cat no. MAK443‐1KT
*Software and algorithms*		
GraphPad Prism version 10 for Windows	GraphPad software	www.graphpad.com
*Other*		
Dynabead's human T‐activator CD3/CD28 for T cell activation	GIBCO Life Technologies	Cat no. 11132D
Human CD4^+^ isolation kit	Miltenyi	Cat no. 130‐096‐533
CD4^+^ isolation columns	Miltenyi	Cat no. 130‐042‐401
RPMI, no glucose	Thermo Fisher Technologies	Cat no. 11879020

### 2.6 Extracellular Flux Analysis (Mito Stress Test)

The assay was performed according to the manufacturer's instructions and as we published (Bharath et al. 2020; Zukowski et al. 2023). CD4^+^ T cells were activated for 40 h along with the following treatments ±3NP for SDH inhibition or ± the succinate analogue DES. The activated cells (300,000/well) were plated onto wells of poly‐D‐lysine‐coated Seahorse XF HS mini plate (Agilent, Santa Clara, CA) in extracellular flux assay media (non‐buffered DMEM containing 5 mM glucose, 2 mM L‐glutamine, and 1 mM sodium pyruvate). Oxygen consumption rate (OCR) was measured as technical duplicates for each donor for each treatment using the mitochondrial stress test procedure for basal OCR, followed by sequential addition of 3.5 μM oligomycin (Calbiochem, San Diego, CA), 1 μM fluoro‐carbonyl cyanide phenylhydrazone (FCCP) (Enzo Life Sciences, Farmingdale, NY), and 14 μM antimycin A (Enzo Life Sciences) with the XF HS mini Extracellular Flux Analyzer (Agilent, Santa Clara, CA) as previously described (Bharath et al. 2020; Nicholas et al. 2019). The data were normalized to cell number.

### 2.7 Lactate Assay

Cellular lactate amounts were assessed utilizing a commercial fluorometric lactate assay performed according to the manufacturer's instructions.

### 2.8 Mitochondrial Network Analysis (MiNA)

Mitochondrial network analysis was performed, and images were processed as described (Valente et al. 2017). Mitochondrial branching was assessed using the Mitochondria Analyzer in ImageJ/Fiji. Mitochondrial network analysis was performed on samples processed as described in the immunofluorescence section. Briefly, cells were incubated with an antibody against the mitochondrial outer membrane protein TOM20. Single‐cell images from well‐resolved mitochondrial structures were obtained. Images obtained with identical settings across various treatments using the Zeiss LSM 800 microscope were processed to produce a binary, skeletonized network as described (Valente et al. 2017). Mitochondria Analyzer, an ImageJ/Fiji plugin, was used to analyze the mitochondrial structure. Mitochondrial branching was normalized to cell size. The three‐dimensional Z‐stacked images were analyzed using IMARIS software.

### 2.9 IMARIS

Mitochondrial volume (μm^3^) was measured using the surface tool in IMARIS with Z Stack 64× images stained for mitochondrial TOM20. All images were deconvolved to ensure accurate surface identification. A smooth surface was set to 0.031 to smooth the image's grain. Background subtraction (local contrast) was used to detect mitochondria. One background‐subtraction parameter was used for all images; each image was then inspected for surface accuracy, and manual adjustments were made as needed. To quantify mitochondrial area, we applied standard deconvolution in IMARIS to the green channel (TOM20). Following deconvolution, we used the IMARIS background‐subtraction protocol and measured the diameter of the largest surface in each image to define the segmentation parameters. These parameters guided the software in identifying which structures to measure (surface area and volume). Manual adjustments were then performed for each image to ensure that IMARIS accurately segmented only the TOM20‐positive (green) structures and that volume measurements remained confined within the green signal boundaries.

To eliminate bias and standardize measurements across donors, only measurements of positively identified mitochondria were used. Additionally, lower and upper thresholds were set, and only measurements within those confinements were used.

### 2.10 Cell Viability

Cell viability was assessed via trypan blue exclusion according to the manufacturer's directions after the different treatments.

### 2.11 Cytokine Assay

Cytokine production was assessed in supernatants by bioplex using a Milliplex human Th17 25‐plex kit (Millipore), as we published (Ip et al. 2016; Conway et al. 2022; Bharath et al. 2020; Zukowski et al. 2023). Outcomes from wells with < 35 beads read per analyte were excluded from the analysis. Samples with > 10% CV were removed from analysis. Plates were washed between incubations using a BioTek 406 Touch plate washer (BioTek) and read using the BioRad FlexMap 3D system (Luminex). Alternatively, cytokine production was assessed by ELISA. Legend Max IL17A/F heterodimer assay (Biolegend) was utilized, and the assay was performed according to the manufacturer's instructions.

### 2.12 scRNA‐Seq Analysis: Differential Expression Analysis of TCA Cycle Genes

The scRNA‐seq libraries were prepared using Reagent Kits (version 3.1, PN‐1000121) according to the Chromium Next GEM Single Cell 3′ protocol from 10× Genomics. Cells were loaded onto chip G to target a recovery of 10,000 cells per sample. Each library was sequenced on an Illumina NextSeq 2000 platform at a depth of 20,000 reads per cell. For the scRNA‐seq libraries, CellRanger (v.6.0.1) was used for sample‐demultiplexing, barcode processing, and single‐nuclei gene‐UMI counting, resulting in a total of 54,078 cells. After initial quality control to remove low‐quality cells and potential doublets, 39,227 cells were retained for downstream analysis.

The expression of TCA cycle genes was compared between cells from younger and older adults using the Wilcoxon rank‐sum test. The analysis was performed separately for all cells and for the Th17 cell subset. Log fold changes (logFC) and the corresponding −log_10_ (*p*‐values) were visualized using waterfall plots generated with the SeuratExtend R package (Hua et al. 2024). In this analysis, logFC was computed using the natural logarithm (base e) with a pseudocount added to both the numerator and denominator to avoid division by zero and to stabilize calculations for low expression values. Prior to logFC computation, normalized expression data were transformed using the expm1 function to ensure consistency with Seurat's FindMarkers function. Genes were ranked by logFC values, where negative values indicate down‐regulation in the young group and positive values indicate up‐regulation.

### 2.13 Partial Least Squares Modeling

Interpolation of cytokine concentrations was performed using a 5‐point logistic curve in Xponent (Luminex). Technical replicates outside of the interquartile range by more than 50% of the mean value were considered outliers and removed. Following these steps, cytokines were not considered for further analysis if more than half of the assayed samples had amounts below the limit of quantification.

Partial least squares discriminant analysis (PLSDA) was used to identify signatures of analytes covarying with outcomes (e.g., treatment group and control). Data were mean‐centered and unit‐variance scaled prior to modeling. PLSDA models were built in R using the *ropls* package (Thévenot et al. 2015). Cross‐validation test sets were sampled as a random 1/3 of the dataset and resampled 100 times or the maximum number of unique test sets, whichever is fewer, to prevent overfitting artifacts. The number of latent variables was chosen based on the highest cross‐validation accuracy.

We calculated the statistical significance of our model using a permutation test. Briefly, the group identities of samples were randomly shuffled in order to preserve the landscape of the data, but with random associations. Cross‐validation was performed using the same number of LVs from the “real” model to estimate true random accuracy based on the preserved data landscape. One hundred randomized models were created to form a distribution of accuracies. The *p*‐value of the “real” model was calculated by comparison of the mean and standard deviation of the distribution of the random models' accuracy.

Models in this study were orthogonalized to maximally project the covariation of the measured cytokines with the response of interest to the first latent variable.

Key cytokines were identified using their Variable Importance in Projection (VIP) score, a measure of a variable's normalized contribution to the predictive accuracy of the model across all considered latent variables. VIP score > 1 indicates a greater‐than‐average contribution to the model, which we used to define the cytokine signature of a specific treatment group.

### 2.14 Statistical Analysis

Data are presented as mean ± standard error of the mean (SEM). Depending on the data set, different statistical tests were applied. The Mann–Whitney *U* test, the Wilcoxon Rank Sum test, and the Kruskal–Wallis test with Dunn's post hoc test or one‐way ANOVA with Bonferroni post hoc tests were used to compare means. Significance was accepted when *p* < 0.05. Robust regression and Outlier removal, the ROUT method, was used to detect outliers. Graph‐Pad Prism 10.0 (GraphPad Software) was utilized for statistical analysis and graphing. We also used partial least squares as a multivariate statistical modeling technique because cytokines have highly interacting regulatory pathways, with their signaling pathways activating transcription factors that amplify the expression of other cytokines, making statistics on individual cytokines incomplete and potentially over‐ or underestimating the relationships of different cytokines to outcomes. We use this multivariate method to obtain a complete, systems‐level view of the complex relationship between the immune milieu and the conditions in each of our experimental groups. In this way, we are able to identify a “signature” that is nuanced, refined, and correctly statistically considered as the covariation of multiple species, allowing a more specific definition of the immune response than a single cytokine can provide.

## 3 Results

### 3.1 SDH Protein Expression Was Higher in CD4 ^+^ T Cells in Older Adults

**FIGURE 1:**
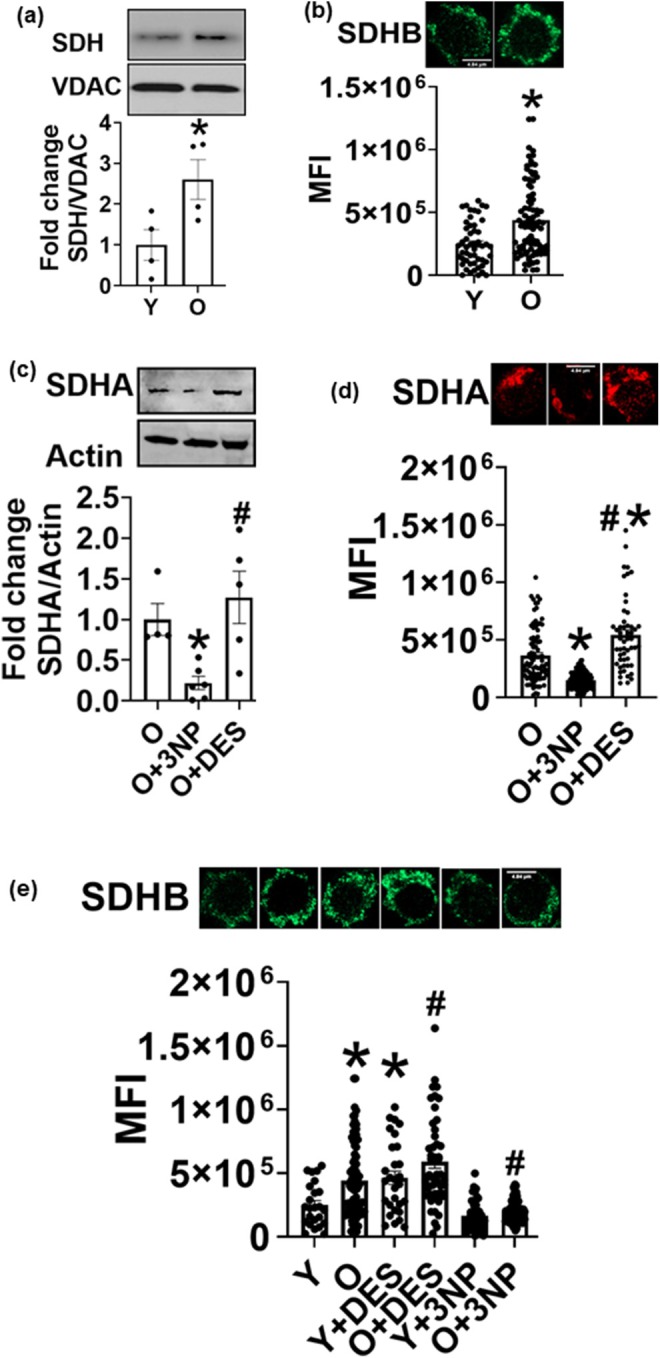
Succinate dehydrogenase protein expression increases with age in CD4^+^ T cells. SDH expression in young (Y) and older (O) adults assessed via immunoblotting (a) and confocal microscopy (b). SDHA protein expression in CD4^+^ T cells treated with SDH inhibitor 3‐nitropropionic acid (3NP) and cell‐permeable diethyl succinate (DES); immunoblotting (c) and microscopy (d). SDHB protein expression after the different treatments (e). *N* = 3–4 (a–e). *N* = 3–4 indicates cells were obtained from either three or four individuals for each condition. Microscopy data are represented as cells in the field of view. At least 5–7 fields per slide were imaged at 63× magnification with oil immersion, on a Zeiss LSM 800 confocal microscope. In fields where numerous cells were observed, the mean fluorescence intensity of 3–4 cell groups was plotted as a single dot. Images were processed as described in the methods, and brightness was adjusted to improve clarity. Mann–Whitney Test or Kruskal–Wallis test with Dunn's post hoc test, *p < 0.05 vs. Y. ^#^ *p* < 0.05 vs. O or O + 3NP.

The expression of mitochondrial complex II, SDH, was higher in CD4^+^ T cells from older adults compared to younger adults (Figure 1a,b). 3‐nitropropionic acid (3NP), a pharmacological SDH inhibitor that irreversibly binds to the active site of the enzyme (Alston et al. 1977), reversed age‐induced increased protein expression of SDH. 3NP reduced the expression of both SDHA and SDHB subunits, which was assessed by immunoblotting (Figure 1c), and by confocal microscopy, SDHA (Figure 1d) and SDHB (Figure 1e). We also tested two other inhibitors of SDH, Atpenin 5 and dimethyl malonate (DMM). DMM compromised cell viability, and 3NP caused a robust accumulation of succinate compared to Atpenin 5 (data not shown). Thus, we used 3NP for subsequent studies. T cell activation markers CD69 and CD25, and cell viability remained unchanged upon 3NP treatment (Figure S3a–c). In our experiments, diethyl succinate (DES, a cell‐permeable form of succinate) did not increase cellular succinate levels but instead increased SDH expression (Figure 1c–e). Diethyl succinate is cleaved by esterase to produce succinate, which can then be rapidly oxidized by SDH. SDH upregulation is likely due to substrate‐driven metabolic adaptation. A rapid increase in intracellular succinate increases flux through SDH, and cells respond to this substrate availability by increasing SDH. It is also important to note that succinate is a potent intracellular signaling molecule that can activate signaling pathways and increase the levels of SDH subunits. Activated T cells exhibit high metabolic flux and can respond more rapidly to metabolic perturbations. There are no well‐established genetic gain‐of‐function methods to activate SDH. DES treatment did not alter CD69 expression (Figure S3a); however, CD25 expression was lower when compared to untreated control (Figure S3b). DES did not affect cell viability (Figure S3c). Cell viability and activation markers were assessed after the completion of 40 h of incubation.

### 3.2 Inhibition of SDH in T Cells From Older Adults Promotes Morphological Changes in the Mitochondria That Resemble Those in T Cells From Younger Adults

**FIGURE 2:**
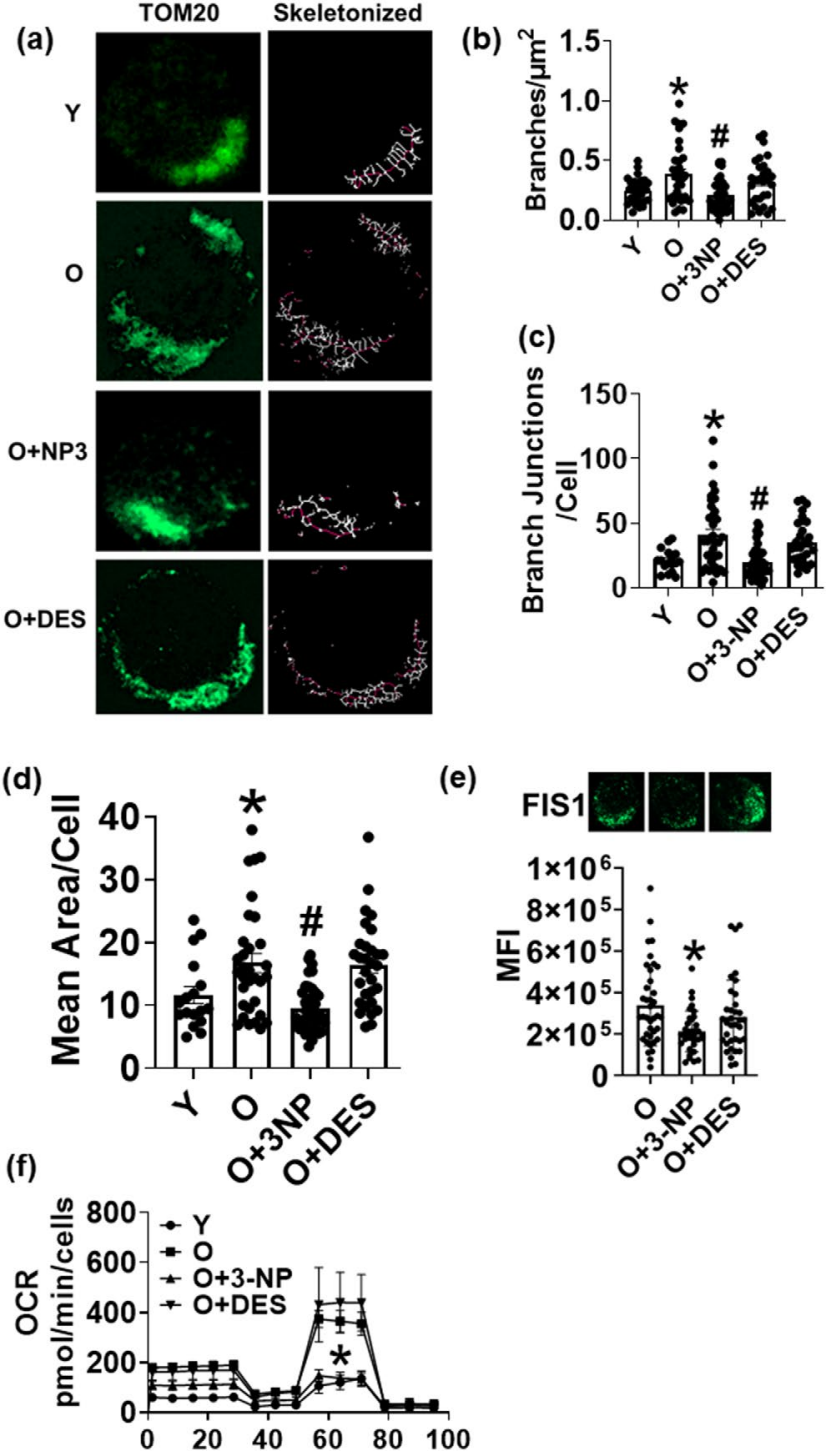
Pharmacological inhibition of SDH alters mitochondrial structure and function. Mitochondrial network analysis was performed on T cells from young (Y) and older (O) adults. Representative images (a) branches (b) branch junctions (c). Mean area (d). Outer mitochondrial fission protein (Fis 1) expression (e). Mitochondrial oxygen consumption rate (f). *N* = 3–4 (a–e) and *N* = 4–8 (f), adults in each group. For microscopy, at least 5–7 fields per slide were imaged at 63× magnification with oil immersion, on a Zeiss LSM 800 confocal microscope. Images were processed as described in the methods, and brightness was adjusted to improve clarity. Mitochondrial branching was quantified per cell and normalized to cell area. One‐way ANOVA with Bonferroni's post hoc or Kruskal–Wallis test and Dunn's post hoc test was performed according to the data set, *p < 0.05 vs. Y or vs. O in panel e, ^#^ *p* < 0.05 vs. O.

We assessed changes in mitochondrial morphology in T cells from younger and older adults following SDH manipulation with 3NP or DES. Morphological changes were evaluated in 2D using Mitochondria Analyzer in ImageJ. T cells from older adults had a branched morphology (Figure 2a, representative image; Figure 2b, quantification), with more junctions per cell (Figure 2c) than T cells from younger adults. An increase in the mean area of mitochondria was also observed in T cells from older adults (Figure 2d). The inhibition of SDH in T cells from older adults reduced mitochondrial branching, branch junctions, and area, similar to T cells from younger adults (Figure 2b–d, 1st bar vs. 3rd bar). The expression of mitochondrial fission protein Fis 1 (Figure 2e) was lower in T cells from older adults with SDH inhibition. To evaluate if the morphological changes of the mitochondria also resulted in functional changes after inhibition of SDH, we performed the mitochondrial stress test. As reported by us previously, the oxygen consumption rate of T cells from older adults was significantly higher than that of T cells from younger adults (Bharath et al. 2020). SDH inhibition with 3NP prevented age‐induced increase in oxygen consumption (Figure 2f and Figure S1a,b). The proton leak and ATP‐linked respiration in T cells from older adults after SDH inhibition were not different when compared to T cells from younger adults (Figure 1c,d). Cellular lactate levels did not change with SDH inhibition at the time point we tested (Figure S1e). Collectively, these data indicated that SDH inhibition altered age‐associated morphological and functional changes in older adults' T cell mitochondria, resembling T cell mitochondria from young adults.

### 3.3 SDH Activation Is Important for Th17 Cytokine Production in T Cells During Aging

**FIGURE 3:**
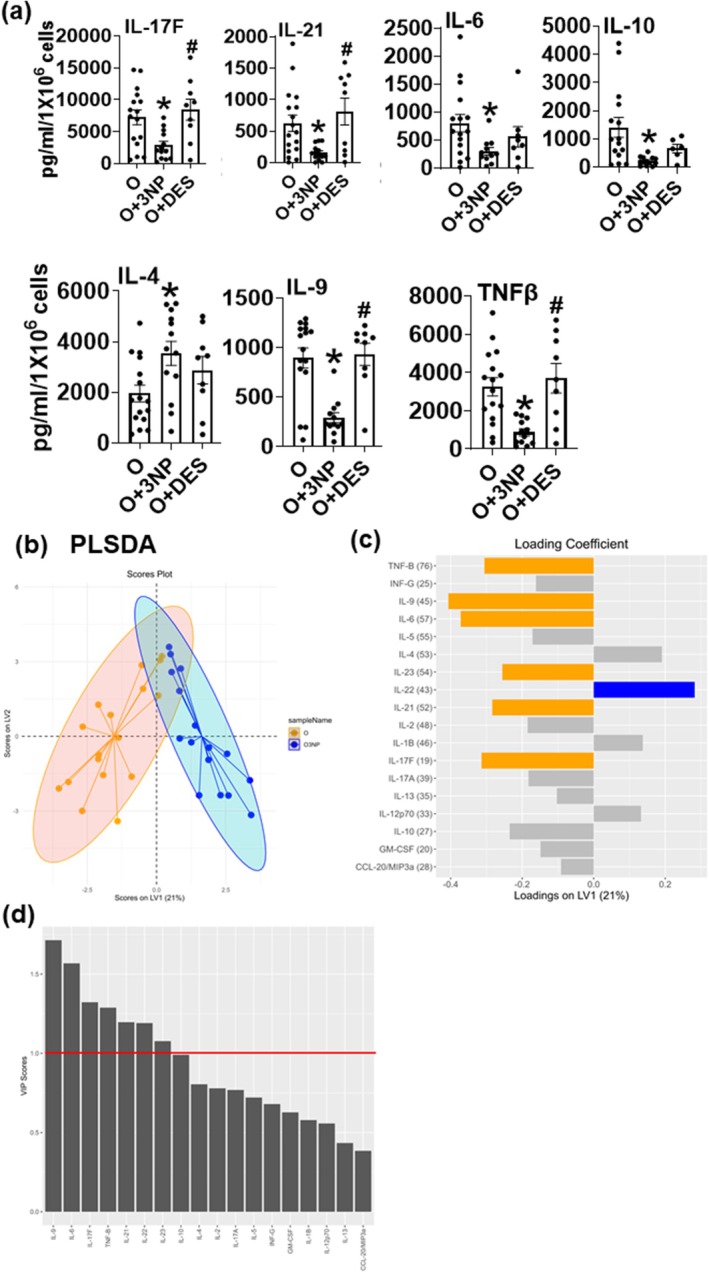
SDH inhibition alleviates Th17 cytokine production in T cells from older adults. A Luminex bioplex assay quantified cytokine production in T cells from older (O) adults ±3NP ± DES (a) PLS‐DA scores plot (2 LV, accuracy: 89%, confidence: 95%) (b). Yellow cytokine loadings signify cytokines with a VIP score > 1 and thus most influential in distinguishing the experimental groups (c). Variable importance projection scores (VIP) (d). Each point represents a single individual in (b). *N* = 12–15 (a–d) adults in each group. Kruskal–Wallis and Dunn's post hoc test was performed for univariate analysis. *p < 0.05 vs. O, ^#^ *p* < 0.05 vs. O + 3NP.

Our earlier work showed CD4^+^ T cells from older adults produced higher amounts of cytokines that are generally considered proinflammatory such as IL‐17A, IL‐17F, IL‐21, TNFα, and IL6 (Bharath et al. 2020). As demonstrated in our previous work (Ip et al. 2016; Nicholas et al. 2019; Wood et al. 2015), we find high levels of covariation in cytokine expression because of the highly interacting pathways of downstream signaling, triggering increased gene expression. Because of this high covariation, individual cytokine levels are not independent variables and cannot be treated with univariate statistics. We thus applied a multivariate statistical modeling technique, partial least squares discriminant analysis (PLSDA, Methods), to identify quantitative patterns of covarying cytokine secretion that distinguish treatment groups. We found that 3NP‐mediated inhibition of SDH in T cells from older adults significantly suppressed our previously defined inflammaging signature, decreasing cytokines such as IL‐17F, IL‐21, IL‐6, IL‐10, IL‐9, and TNFβ, whereas promoting the production of the Th2 cytokine IL‐4, which can inhibit Th17‐supportive IL‐6 (Figure 3a). Other cytokines changed differentially (Figure S2a). In light of the highly interacting nature of cytokine signaling and immune regulation, it is important to note that PLSDA identifies a pattern of cytokines that co‐vary, indicating their importance as a signaling module, and the pattern itself is the entity by which we evaluate both biological and statistical significance. This pattern of altered cytokine secretion differentiates O and O3NP samples with 89% accuracy and 95% confidence (Figure 3b). On the loadings plot (Figure 3c), colored bars are analytes that contributed more than average to the predictive accuracy of the model (VIP score > 1, Figure 3d). We conclude that SDH is important for a Th17 proinflammatory phenotype in T cells during aging, and preventing age‐induced hyperactivation of SDH would normalize the profiles observed in T cells from older adults.

Although we observed a significant decrease in CD25 expression upon DES treatment at 40 h, we did not observe a decrease in the production of cytokines that are altered with age. Many effector cytokines, including IL‐17, are TCR and transcription factor‐driven and not IL‐2 dependent once activation is initiated. Thus, the cells continue to produce cytokines even if they are less responsive to IL‐2.

### 3.4 Genetic Inhibition of SDHA Reversed Age‐Induced Mitochondrial Structural Changes and Th17 Cytokine Production in T Cells From Older Adults

**FIGURE 4:**
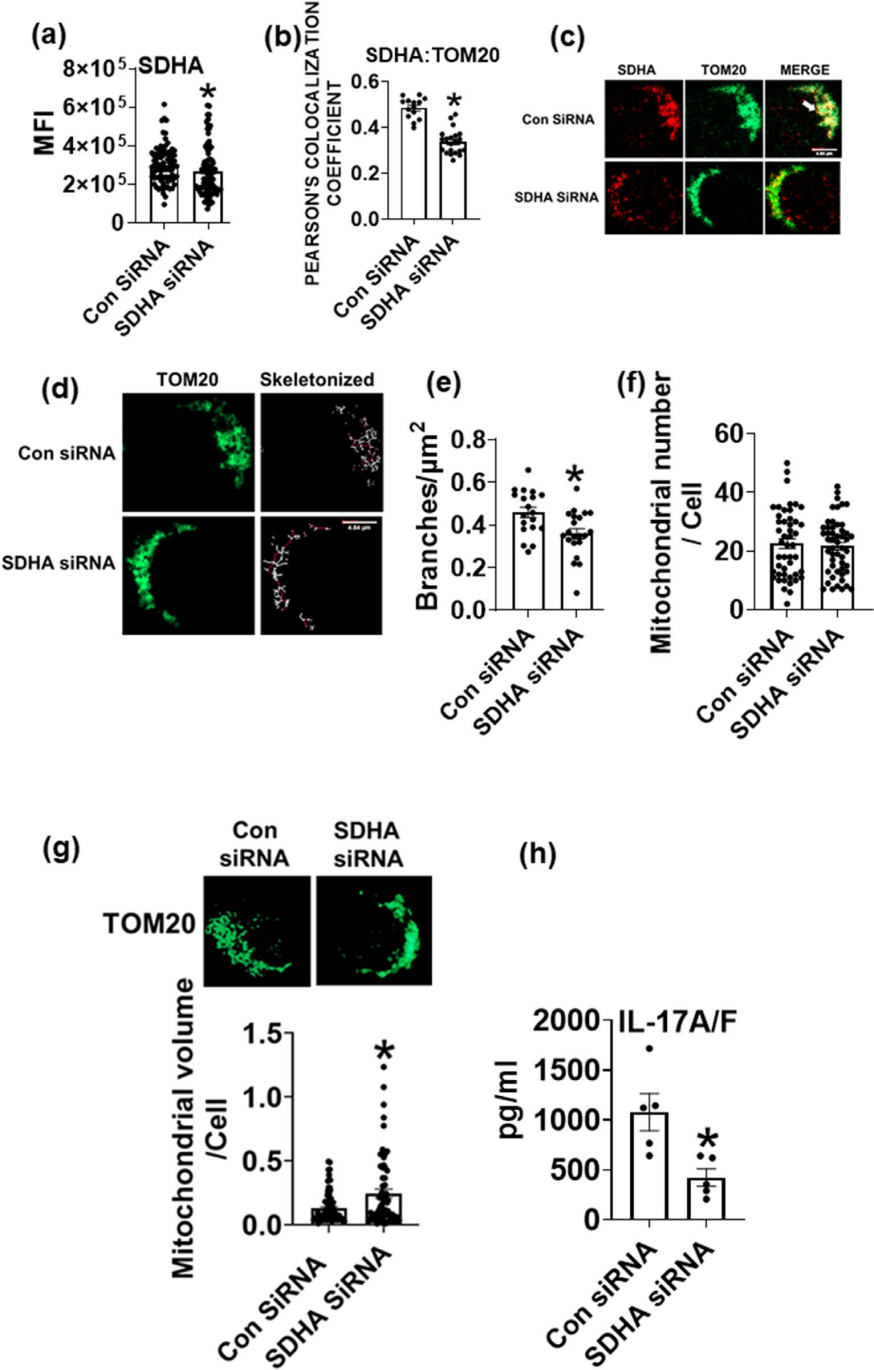
Genetic inhibition of SDHA prevents age‐induced mitochondrial structural changes and Th17 cytokine production in T cells from older adults. Quantification of SDHA protein expression after siRNA‐mediated knockdown of SDHA (a) Quantification of localization of SDHA with mitochondrial protein TOM20 (b) representative images (c). Representative images of mitochondrial network analysis performed on T cells ± SDHA siRNA (d) quantification of branches (e) mitochondrial number (f). 3D IMARIS analysis of mitochondrial volume (g) IL17A/F heterodimer levels after SDHA siRNA, assessed via ELISA assay (h). *N* = 3–5 (a–h) adults in each group. Mann–Whitney test. *p < 0.05 vs. Con siRNA.

Next, we assessed mitochondrial structure and cytokine production after genetic inhibition of SDH. siRNA to the subunit A of the SDH reduced SDHA protein amounts (Figure 4a) and SDHA localization within the mitochondria (Figure 4b,c, representative images) compared to scrambled control siRNA. T cells treated with SDHA siRNA had fewer mitochondrial branches/cell (Figure 4d; representative image and Figure 4e; quantification), with no change in mitochondrial number (Figure 4f). Higher‐resolution analysis using IMARIS 3D revealed that T cells from older adults treated with SDHA siRNA had higher mitochondrial volume than control siRNA‐treated cells (Figure 4g). IL‐17A/F heterodimer cytokine production assessed via ELISA showed a significant reduction in SDHA siRNA‐treated cells compared to con siRNA (Figure 4h).

### 3.5 Overactive SDH Promotes Stabilization of HIF‐1α to Promote Th17 Cytokines in Aging

**FIGURE 5:**
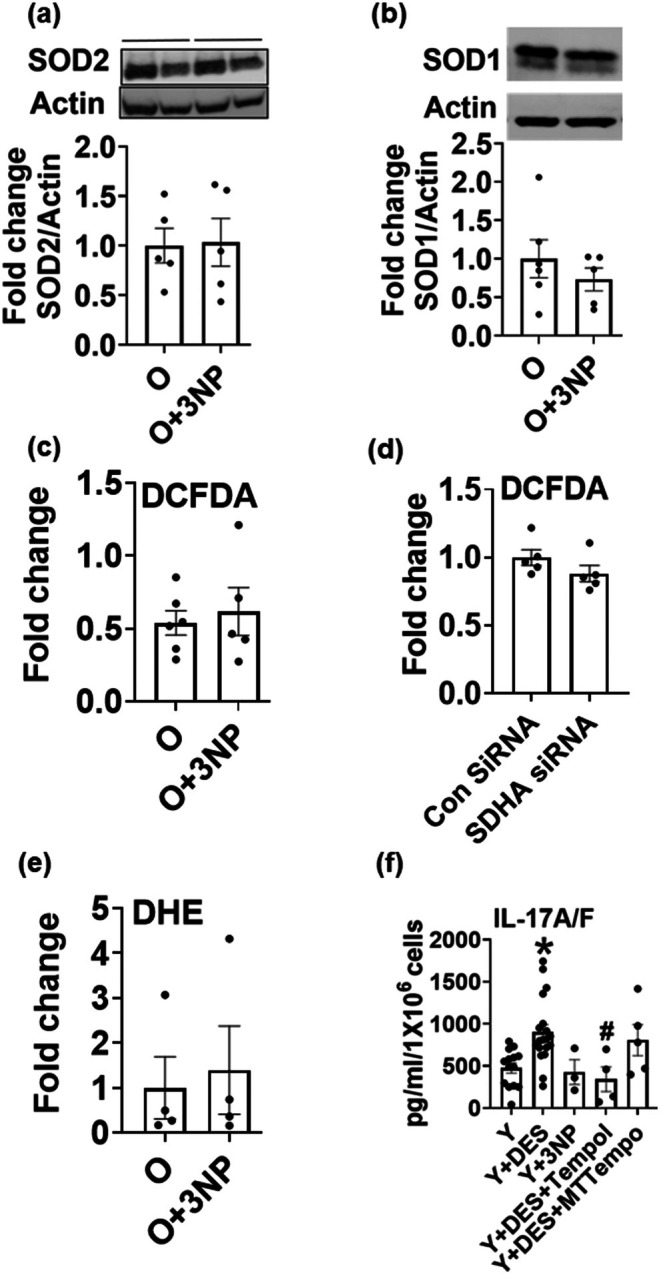
Cellular redox balance is not altered by SDH inhibition. Protein expression of mitochondrial (SOD2) (a) or cytosolic (SOD1) (b) ROS regulatory enzymes. H_2_O_2_ assessed after pharmacological (c) or genetic (d) inhibition of SDH. Cellular superoxide (O_2_^−^) after SDH inhibition (e). Cytokine production assessed via an ELISA assay in T cells from Y adults ± DES ± ROS scavengers (f). *N* = 5 (a–e), *N* = 4–12 (f), adults in each group. Mann–Whitney test or Kruskal–Wallis test with Dunn's post hoc test was performed. *p < 0.05 vs. Y, ^#^ *p* < 0.05 vs. Y + DES.

To gain a mechanistic understanding of how SDH regulates Th17 inflammation, we evaluated mitochondrial and cellular redox balance, since reactive oxygen species (ROS) play a vital role in the induction of Th17 inflammation (Zhi et al. 2012; Chávez and Tse 2021). SDH inhibition with 3NP did not alter the expression of mitochondrial ROS regulatory enzyme superoxide dismutase 2 SOD2 (MnSOD) (Figure 5a) or cytosolic superoxide dismutase SOD1 (Cu/ZnSOD) (Figure 5b). ROS assessed via DCFDA fluorescence showed no change after pharmacological (Figure 5c) or genetic SDH inhibition (Figure 5d). No change was observed in dihydroethidium (DHE) fluorescence, which measures cellular superoxide amounts (Figure 5e). Despite no indication of 3NP‐mediated changes in ROS, Tempol, which scavenges ROS (among other functions), reduced the production of heterodimeric IL17A/F in T cells from Y adults treated with DES, whereas mitochondria‐targeted ROS scavenger Mitotempo (MTTempo) did not (Figure 5f).

**FIGURE 6:**
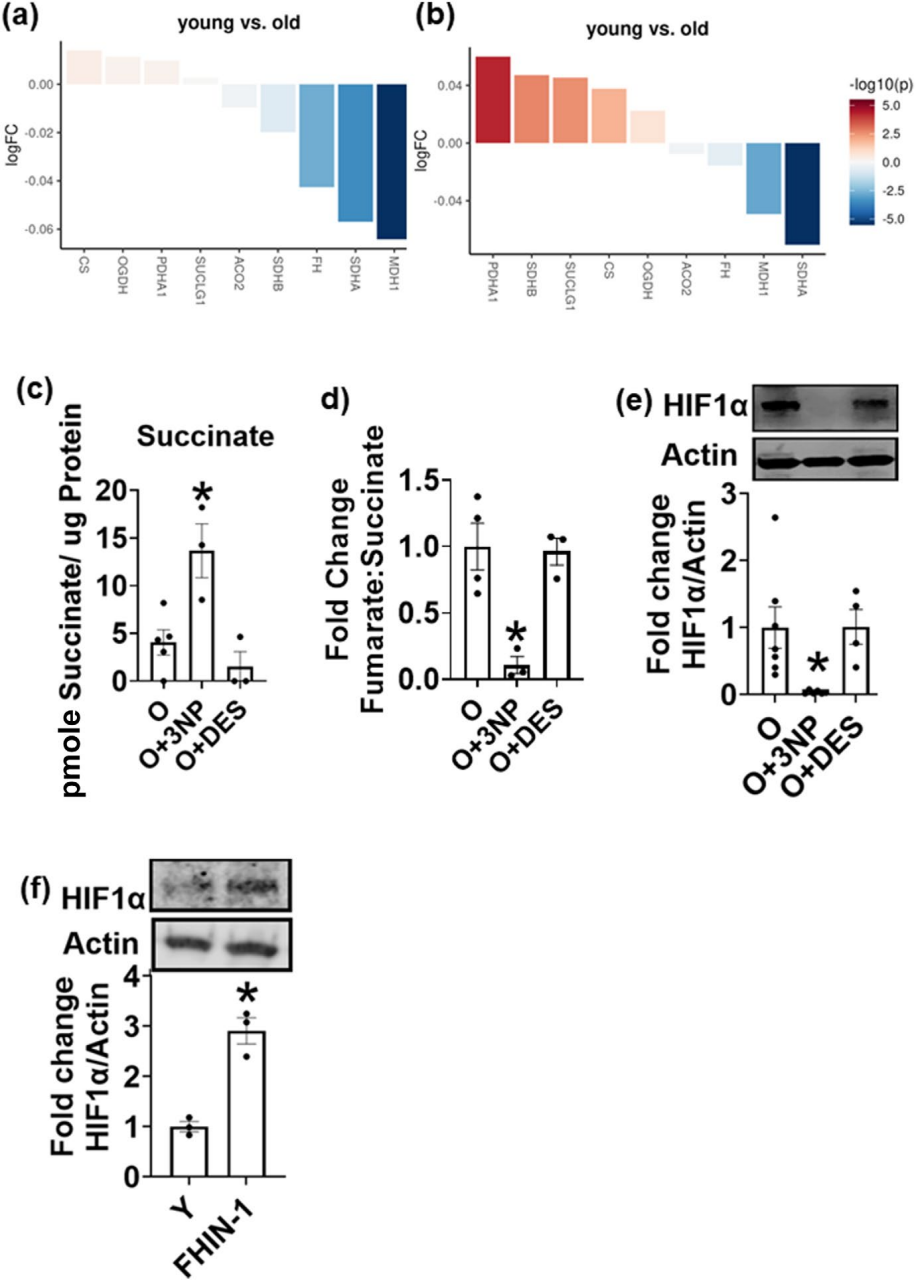
Age‐induced dysregulation of TCA cycle metabolites fuel Th17 cytokine production. Waterfall plots showing ScRNA seq analysis of TCA cycle enzymes in CD4^+^ T cells from young (Y) and older (O) adults (a) ScRNA seq analysis of TCA cycle enzymes in Th17 subset of T cells (b). Cellular amounts of succinate (c) fumarate:succinate ratio (d) HIF1α protein in T cells from older adults (e) and HIF1α protein in T cells from younger adults after FH inhibition (f) *N* = 3, (a, b) *N* = 3–4, (c, d) *N* = 5–7, (e) *N* = 3, (f) adults in each group. One‐way ANOVA with Bonferroni test or Wilcoxon matched‐pair signed rank test. *p < 0.05 vs. O or Y.

Since SDH inhibition did not impact major cellular ROS species, we tested whether changes in metabolites could impact the induction of Th17 inflammation with age using single‐cell RNA sequencing. Compared to CD4^+^ T cells from older adults, the T cells from younger adults expressed less SDHA, SDHB, fumarate hydratase (FH), and malate dehydrogenase (MDH1) (Figure 6a). Given the parallel roles of SDH in the TCA cycle, we evaluated the transcripts of the TCA cycle enzymes in Th17 clusters, identified using Th17‐preferential genes CD4^+^, CCR6^+^, CCR4^+^, IL17^+^, and KLRB1^+^ as we published (Zukowski et al. 2023). Compared to CD4^+^ T cells from older adults, the T cells from younger adults had significantly less SDHA (Figure 6b). Collectively, the data points to likely dysregulation of the TCA cycle with age. To test this possibility, we assessed the cellular levels of the TCA metabolites to pinpoint mechanisms that link SDH and Th17 inflammation. We quantified succinate and fumarate in CD4^+^ T cells from older adults after treatment with 3NP and DES. Succinate significantly increased upon SDH inhibition with 3NP, whereas DES had no effect on succinate levels within the cell (Figure 6c). The ratio of fumarate to succinate was dramatically lower in T cells treated with 3NP (Figure 6d).

Perturbations in TCA cycle enzymes that alter metabolite balance can promote cytosolic signaling events that may affect cytokine production (Lampropoulou et al. 2016; Mills et al. 2016). Fumarate is known to stabilize the transcriptional activator HIF1α by preventing its proteosomal degradation (Isaacs et al. 2005), which in turn could increase the production of Th17 cytokines and IL‐6. HIF1α can act as a transcriptional regulator of IL‐6 and can aid in the differentiation of Th17 cells by activating RORC, the master transcription factor for Th17 lineage. We observed that the HIF1α in T cells from older adults was completely abrogated in the presence of 3NP (Figure 6e). To test if fumarate levels are linked to HIF1α activation, we pharmacologically inhibited fumarate hydratase (FH), an enzyme that converts fumarate to malate, in T cells from young adults and assessed HIF1α. Inhibition of FH induced robust H1F1α (Figure 6f). We conclude that age‐induced hyperactivation of SDH alters the levels of succinate and fumarate, which, in turn, influence HIF‐1α and, the production of proinflammatory cytokines that increase during physiological aging.

## 4 Discussion

In this study, we sought to understand the mechanistic regulation and identify the key contributors to Th17 inflammation during physiological aging. Although our previous work indicated that mitochondrial STAT3 plays a vital role in Th17 inflammation, mathematical modeling predicted the involvement of other unknown player(s) that impact the ability of mitochondrial STAT3 to regulate T cell‐mediated inflammation. To our knowledge, we are the first to show that SDH is critical for Th17 cytokine production during physiological aging. Our data showed that inhibition of SDH significantly altered succinate:fumarate levels in T cells from older adults, restoring mitochondrial morphology to that observed in T cells from younger adults and alleviating Th17 cytokine production. Collectively, our data provide evidence for the existence of mechanistic regulation between SDH and Th17 cytokine production in aging.

SDH is important for ATP generation and plays a major role in numerous intracellular signaling pathways, in part, through its product, succinate (Murphy and Chouchani 2022). Succinate is an immunomodulatory molecule and an oncometabolite (Wang et al. 2025). Succinate can regulate intracellular signaling and can also be transported to the extracellular space to act as an extracellular signaling molecule (Huang et al. 2024). Succinate transporters facilitate the efflux and influx of succinate, thereby dynamically maintaining its levels within both the mitochondria and the cell.

A highly branched and reticular mitochondrial arrangement is generally associated with heightened OXPHOS, as we observed in T cells from older adults (Zukowski et al. 2023; Leduc‐Gaudet et al. 2015; Glancy et al. 2020). Mitochondria exist in a dynamic state, undergoing fission and fusion, and forming more complex arrangements and branching. Mitochondrial morphology correlates with T cell differentiation, T cell subsets, activation, and functional status. Promoting mitochondrial fusion by overexpression of opa1 promotes cristae remodeling, memory cell formation, and antitumor activity (Buck et al. 2016). A strong correlation also exists between the metabolites of the mitochondria and its morphology. Metabolite‐mediated redox status of the cell is well known to actively regulate mitochondrial network, function, and morphology (Singh et al. 2025). SDH upregulation in older adults' T cells resulted in lower succinate levels due to rapid oxidation of succinate to fumarate. Interestingly, we did not observe a concomitant upregulation of fumarate hydratase (FH) in T cells from older adults, which indicated that FH is not metabolizing the incoming fumarate at the same rate as SDH thus resulting in a higher fumarate: succinate ratio. Recent work in cells and in a transgenic mouse model shows that a mutation in FH resulted in the accumulation of fumarate, which induced progressive and robust remodeling of mitochondrial morphology, activation of proinflammatory pathways and induction of tumorigenesis (Zecchini et al. 2023). The chronic model of FH loss also resulted in changes in mitochondrial morphology. Most importantly, the study indicated that fumarate was the primary driver of mitochondrial network remodeling, whereas succinate had minimal effect and α‐ketoglutarate and 2‐hydroxyglutarate had no effect compared to fumarate (Zecchini et al. 2023). Aging T cells had an imbalance in TCA cycle metabolites with higher fumarate due to changes in the fumarate: succinate ratio, which likely contributed to the changes in mitochondrial morphology through alteration of mitochondrial dynamics. Restoration of the fumarate: succinate ratio by inhibition of SDH resulted in mitochondrial structural (reduced branching) and functional phenotypes (reduced OCR and proton leak) that recapitulated those of T cells from young adults. However, cellular lactate production, an indicator of changes in glycolysis, was not altered at the time point we tested.

Our data also showed that the changes in succinate and fumarate might be intricately involved in the regulation of T cell cytokine production because SDH inhibition significantly and specifically reduced the production of Th17 (IL‐17 F, IL‐21, IL‐23) and IL‐6 cytokines, thus reversing the age‐associated proinflammatory phenotype. A dysregulation of metabolites, especially succinate and fumarate, promotes a proinflammatory and tumorigenic phenotype in other contexts (Martínez‐Reyes and Chandel 2020). Interestingly, CD4^+^ T cells, as well as the Th17 subsets of T cells from young adults, downregulated the transcripts of SDH, FH, and MDH1. These data collectively indicate that sustaining adequate levels of succinate and maintaining the appropriate ratio of fumarate and succinate might be critical for Th17 cytokine production. Succinate and fumarate play a significant role in immune cell function and inflammation, particularly through their influence on ROS production (Mills et al. 2016; 2018; Cheng et al. 2025). However, SDH inhibition did not show an effect on ROS, although ROS signaling is time‐dependent and highly variable, leaving open the possibility that ROS served as an initial trigger. Thus, we cannot completely rule out the role of ROS in initiating signaling events within the mitochondria or the cell that could trigger age‐related inflammation.

Fine‐tuning of the TCA metabolites in stringent conditions is required to maintain HIF‐1α; a major sensor of metabolic cues within the cell. HIF1α is also an important transcription factor and is known to regulate Th17 differentiation. HIF1α interacts with RORγt and HIF1α deficiency is shown to drastically and specifically inhibit the Th17 pathway (Dang et al. 2011). Although HIF1α is known to be induced under hypoxic conditions, studies have shown that HIF1α is present in normoxia, and HIF1α‐deficient cells in normoxia fail to induce Th17 response (Dang et al. 2011). Factors like ROS, nitric oxide, and metabolites succinate and fumarate are involved in HIF1α signaling in normoxia in a prolyl hydroxylases (PHD) dependent manner.

The enzymes prolyl hydroxylases (PHDs) are oxygen‐dependent dioxygenases that hydroxylate HIF‐1α at its proline 402 and 564 residues and help target HIF1α for degradation by the proteasome. PHDs are degraded under hypoxic conditions, leading to the stabilization of HIF1α during hypoxia. However, PHDs can also be inactivated in normoxia by changes in TCA cycle metabolites such as succinate and fumarate. Succinate and fumarate, which are transported from the mitochondria to the cytosol by the voltage‐dependent anion channel (VDAC) and the dicarboxylate carrier (DIC), can act as competitive inhibitors of PHDs, thereby stabilizing HIF1α under normoxic conditions (Kaelin and Ratcliffe 2008). Higher amounts of cellular succinate could inhibit PHD and thus promote HIF‐1α stabilization. Since we observed abrogation of HIF‐1α despite the increase in cellular succinate levels in T cells from older adults after SDH inhibition (3NP), we concluded that succinate was not stabilizing HIF‐1α in this context.

Fumarate is also known to inhibit PHD enzymes, leading to the stabilization of HIF‐1α (Isaacs et al. 2005). Fumarate acts as a competitive inhibitor of α‐ketoglutarate‐dependent enzymes by competing for the same binding site. Since fumarate was high in older adults' T cells, where robust HIF‐1α was observed, we concluded that fumarate was responsible for HIF‐1α stabilization and the induction of Th17 and IL‐6 cytokines. HIF1α activation in T cells from younger adults after pharmacological inhibition of FH supported our hypothesis that alterations in metabolites affect HIF1α. Metabolite regulation of cellular signaling is highly complex, and many layers of regulation still need to be unraveled. Based on our understanding of the current data, we propose that age‐induced hyperactivation of SDH, altered TCA cycle metabolites succinate and fumarate, promoted mitochondrial structural and functional changes, and impacted Th17 cytokine production. Mitochondrial metabolite‐dependent regulation of HIF1α emerged as important in age‐associated T cell inflammation.

### 4.1 Limitations and Future Studies

Although we propose a mechanism for Th17 inflammation in aging, detailed metabolomics and related mechanisms in T cell inflammation must be elucidated. The evaluation of mechanistic regulation of age‐related inflammation in different subsets of T cells is necessary to gain a comprehensive understanding of the relative contributions. Whether STAT3 interacts directly with SDH or if it influences SDH through signaling is our ongoing investigation. The data presented herein may not be representative of adults older than 80 years of age and those with age‐related comorbidities.

## Author Contributions

Conceptualization – L.P.B. Methodology – L.P.B. Investigation – E.O., G.C., M.N., A.J., L.G., A.L., O.S., K.L., and L.P.B. Formal analysis – E.A.P., J.Y., R.Y.K., and Y.V. Supervision – L.P.B. Writing – L.P.B. Editing – B.S.N., J.M.‐N., J.T.D., H.H., and M.J.D. Funding – L.P.B. Resources – H.H.

## Funding

This work was supported by R15AG068957 (L.P.B.), pilot award from the San Diego Nathan Shock Center of Excellence in the Basic Biology of Aging NIH P30AG068635 (L.P.B.) R56AG06985, R01AG084180, R01AG079525 (B.S.N.), R01AG072513 (E.A.P.) and R01AG076075 (M.J.D.). This work was also supported by the Pasini Fellowship (L.P.B.), Merrimack College, College of Health Sciences, and College of Natural Sciences (L.P.B.) and Barnstable Brown Diabetes Center, University of Kentucky (B.S.N.).

## Disclosure

“The content is solely the responsibility of the authors and does not necessarily represent the official views of the National Institutes of Health.”

## Conflicts of Interest

The authors declare no conflicts of interest.

## Supporting information


**Appendix S1:** acel70451‐sup‐0001‐AppendixS1.pdf.

**Figure S1:** acel70451‐sup‐0002‐FigureS1.tiff.

**Figure S2:** acel70451‐sup‐0003‐FigureS2.tiff.

**Figure S3:** acel70451‐sup‐0004‐FigureS3.tiff.

## Data Availability

The data that support the findings of this study are available from the corresponding author upon reasonable request.
