# Supplementary material for: Role of Succinate Dehydrogenase in Age‐Related Th17 Inflammation
Source: Aging Cell. 2026 Mar 24;25(4):e70451. doi: 10.1111/acel.70451 (PMC13140695; doi:10.1111/acel.70451)
Supplement: Supplementary file 1 — Appendix S1: acel70451‐sup‐0001‐AppendixS1.pdf. [file ACEL-25-e70451-s003.pdf]

AGING CELL AUTHOR CHECKLIST. ***Authors should submit this checklist together with their manuscript. Please ensure that you have read the Author Guidelines in detail before submission.***

Title	**Role of Succinate Dehydrogenase in Age-related Th17 Inflammation**
**Authors**	Evelyn Ocegueda[1#], Gabrielle Chase[2#], Michaella Niceforo[2], Aida Javidan[7], Lydia Gugliuzza[2], Jingting Yu[3,] Rachel Kang[4,], Ava Lankowski[1], Olivia Stefanik[1], Kailey Leclerc[2], Yolander Valentine[7], Micah Drummond[5], Jude T. Deeney[9], Josephine-Modica-Napolitano[2], Elizabeth A. Proctor[4,10], Hatice Hasturk[6], Barbara S. Nikolajczyk[7,8,] and Leena P. Bharath[1*]
**Manuscript Type**	Research Article
**Total Character Count (including spaces)**[1]	47,768
**Word count of Summary**[2]	218
**Number of papers cited in the References**[3]	44
**Listing of all Tables (Table1, Table 2 etc)**[4]	Table 1: Description of Research Subjects<br>Table 2: Identification of Resource

**Figure specifications (please complete one row per figure)**[5]<br><br>***Figure no.***	Colour<br>***(yes/no)***	Greyscale<br>***(yes/no)***	Black and white<br>***(yes/no)***	Single column (80mm)<br>***(yes/no)***	Double column (180mm)<br>***(yes/no)***	Size of figure at full scale (mm x mm)<br>***(insert details)***	Smallest font size used in the figure at full scale (minimum 6pt)<br>***(insert details)***
1	Yes						Arial 20
2	Yes						Arial 20
3	Yes						Arial 9
4	Yes						Arial 20
5	No						Arial 20
6	Yes						Arial 9

[1] The maximum character count allowed is 50,000 (incl. spaces) for Primary Research Papers and Reviews, 10,000 for Short Takes.

[2] Summary should not exceed 250 words.

[3] Primary Research Papers can contain a maximum of two tables. If more are needed they should replace some of the Figures or can be placed in the Supporting Information.

[4] A maximum of 45 references is allowed for Primary Research Papers and 20 references for Short Takes.

[5] A Primary Research Paper may contain up to 6 figures and a Short Take up to 2 figures. Authors are encouraged to provide figures in the size they are to appear in the journal and at the specifications given.
