# Supplementary figures and images for: Role of Succinate Dehydrogenase in Age‐Related Th17 Inflammation
Source: Aging Cell. 2026 Mar 24;25(4):e70451. doi: 10.1111/acel.70451 (PMC13140695; doi:10.1111/acel.70451)

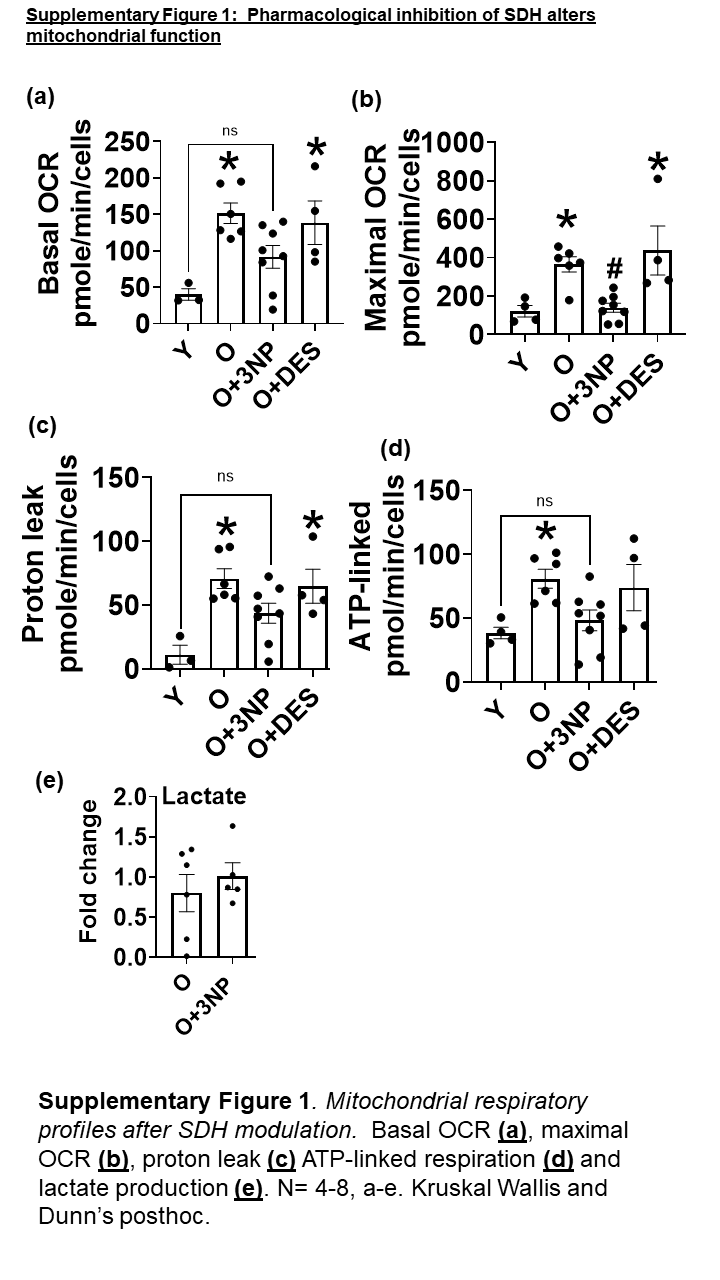

Supplement: Supplementary file 2 — Figure S1: acel70451‐sup‐0002‐FigureS1.tiff. [file ACEL-25-e70451-s004.tiff]

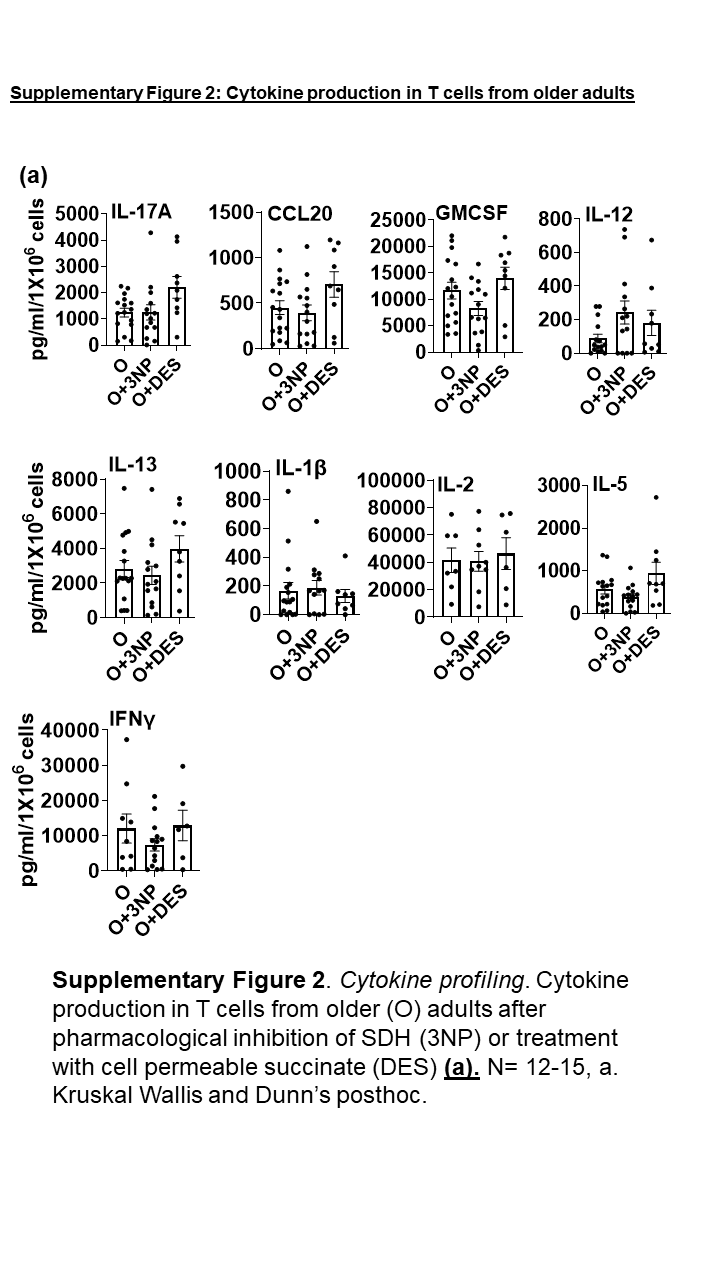

Supplement: Supplementary file 3 — Figure S2: acel70451‐sup‐0003‐FigureS2.tiff. [file ACEL-25-e70451-s002.tiff]

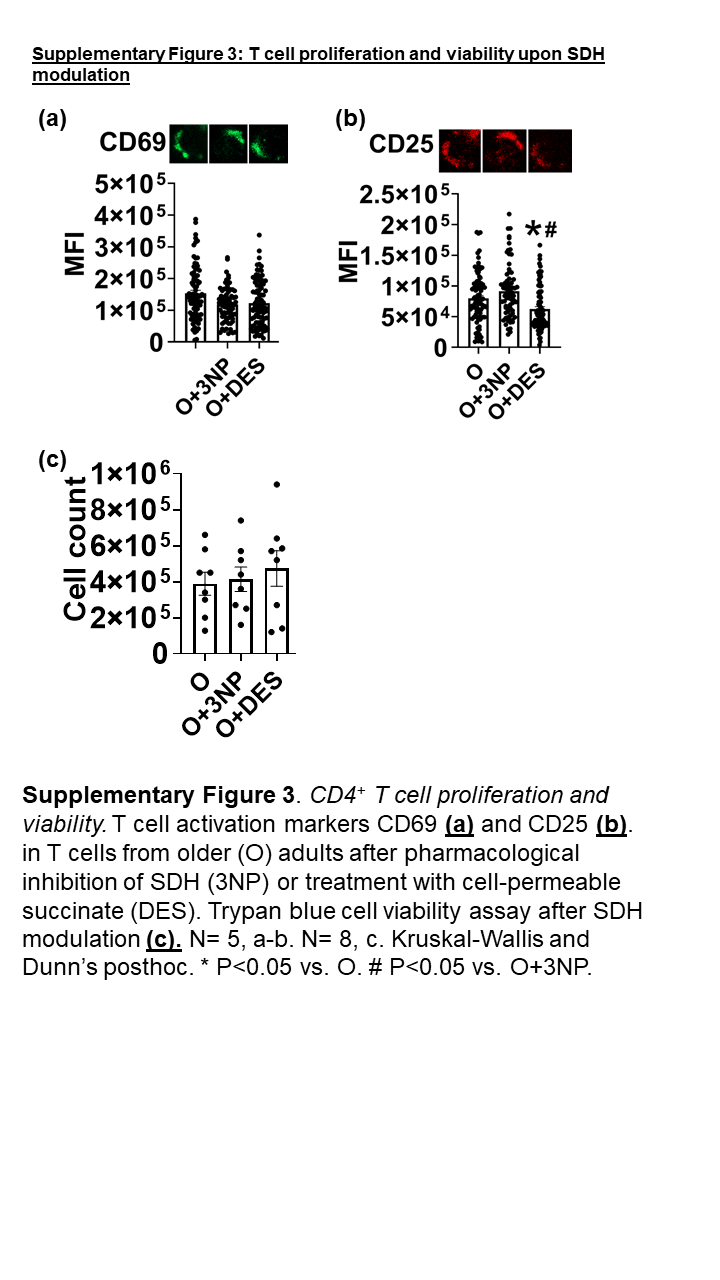

Supplement: Supplementary file 4 — Figure S3: acel70451‐sup‐0004‐FigureS3.tiff. [file ACEL-25-e70451-s001.tiff]
